# The Effect of Three Key Administrative Errors on Patient Trust in Physicians: Prescription Errors, Confidentiality Breaches, and Appointment Scheduling Omissions

**DOI:** 10.1002/jgf2.70086

**Published:** 2025-11-30

**Authors:** Tetsuro Aita, Yoshia Miyawaki, Yu Katayama, Kosuke Sakurai, Nao Oguro, Takafumi Wakita, Nobuyuki Yajima, Ashwin B. Gupta, Noriaki Kurita

**Affiliations:** ^1^ Department of General Internal Medicine and Family Medicine Fukushima Medical University Fukushima City Fukushima Japan; ^2^ Department of Clinical Epidemiology, Graduate School of Medicine Fukushima Medical University Fukushima City Fukushima Japan; ^3^ Department of Nephrology, Rheumatology, Endocrinology and Metabolism Okayama University Graduate School of Medicine, Dentistry and Pharmaceutical Sciences Okayama City Okayama Japan; ^4^ Division of Pathogenesis and Translational Medicine, Department of Clinical Pharmacy Showa Medical University School of Pharmacy Shinagawa‐ku Tokyo Japan; ^5^ Division of Rheumatology, Department of Medicine Showa Medical University Shinagawa‐ku Tokyo Japan; ^6^ Faculty of Sociology Kansai University Suita City Osaka Japan; ^7^ Center for Innovative Research for Communities and Clinical Excellence Fukushima Medical University Fukushima City Fukushima Japan; ^8^ Medicine Service VA Ann Arbor Healthcare System Ann Arbor Michigan USA; ^9^ Division of Hospital Medicine, Department of Internal Medicine University of Michigan Medical School Ann Arbor Michigan USA; ^10^ Department of Innovative Research and Education for Clinicians and Trainees (DiRECT) Fukushima Medical University Hospital Fukushima City Fukushima Japan

**Keywords:** administrative errors, appointment scheduling omissions, confidentiality breaches, prescription errors, trust in physicians

## Abstract

**Background:**

Understanding how administrative errors, such as prescription and appointment scheduling omissions, and patient confidentiality breaches impact trust in physicians is crucial for improving patient‐physician relationships and healthcare outcomes. To investigate the association between administrative errors, general trust in physicians, and interpersonal trust in a physician, we surveyed adults across Japan.

**Methods:**

Participants were adults aged ≥ 20 years who had received treatment at least twice for non‐communicable diseases within the past 6 months. The exposure variables were past experiences with prescription errors, confidentiality breaches, and appointment scheduling omissions by personal physicians treating their non‐communicable diseases. General trust and interpersonal trust in a physician were measured using the Japanese version of the Wake Forest Physician Trust Scale.

**Results:**

Among the 661 participants, nearly 14% reported experiencing at least one type of administrative error. Prescription errors were associated with a significant decrease in general trust in physicians (−9.78 points, 95% confidence interval [CI]: −13.74 to −5.81). Confidentiality breaches had the most significant negative impact on interpersonal trust (−14.09 points, 95% CI: −24.35 to −3.83), followed by appointment scheduling omissions (−13.56 points, 95% CI: −22.48 to −4.65). Mediation analysis revealed that the association between prescription errors and reduced general trust was partially mediated by decreased trust in personal physicians.

**Conclusions:**

Administrative errors during care for non‐communicable diseases significantly undermine patients' trust in physicians. Physicians should prioritize improving their practices, particularly regarding prescription errors, as these errors have broader implications for the public's perception of physicians.

## 1 Introduction

Patient trust in physicians is central to medical care and plays an important role in medication adherence, adoption of recommended preventive behaviors, and ensuring the continuity of care [1, 2, 3]. Trust in physicians can be classified into two constructs: interpersonal trust, which refers to trust in a specific physician, and general trust, which pertains to trust in physicians as a collective group [4, 5]. General trust is particularly important for enabling physicians to gain confidence with new patients who have limited prior interactions [4]. Although numerous studies have shown that medical errors causing physical harm result in diminished trust in the involved physician or institution [6, 7, 8], limited understanding of how administrative errors in outpatient services—errors that do not necessarily involve physical harm—affect both interpersonal and general trust exists.

Prescription errors, for instance, can lead to the delayed recognition of adverse events, causing physical and emotional distress for patients and their families [8, 9]. These errors, whether executive actions (e.g., incorrect prescriptions) or omissions (e.g., failure to prescribe), are known to impact patient well‐being; however, their influence on trust in physicians remains unclear [9]. Similarly, appointment scheduling omissions due to forgetting by medical professionals represent a common administrative omission, yet their impact on trust has not been extensively studied [10]. In addition, patient confidentiality breaches, a cornerstone of the doctor‐patient relationship, pose another critical issue. While confidentiality breaches may not cause direct physical harm, they can erode trust, result in patient humiliation, and lead to broad disadvantages (i.e., legal sanctions) [11, 12]. While it is intuitive that confidentiality violations damage interpersonal trust, their potential to diminish general trust in the healthcare system has not been quantitatively examined [12]. This distinction is critical, as general trust may be impacted differently by these errors than interpersonal trust [4, 5].

In Japan, such administrative errors occur with some frequency, partly due to the complexity of electronic ordering systems and structural challenges in outpatient departments [13, 14]. Physicians often manage both prescriptions and appointment scheduling through these systems without clerical support, making the accuracy highly dependent on individual performance. Moreover, architectural designs such as open ceilings that connect adjacent consultation rooms can inadvertently allow conversations to be overheard, increasing the risk of confidentiality breaches. Addressing these administrative issues should therefore be a priority in primary care and outpatient settings.

Our study aimed to examine the impact of administrative errors—such as prescription errors, patient confidentiality breaches, and appointment scheduling omissions—on both interpersonal trust in their physicians and general trust in physicians as a group among adults with non‐communicable diseases. Furthermore, we explored whether interpersonal trust in their physicians mediates the relationship between these errors and general trust, offering insights into the importance of different dimensions of trust in healthcare management.

## 2 Methods

### 2.1 Study Design and Subjects

This cross‐sectional study used an online panel survey conducted through Cross Marketing, a web‐based company located in Shinjuku‐ku, Tokyo, to recruit Japanese participants with non‐communicable diseases. The survey was conducted on April 27 and 28, 2020, targeting individuals aged 20 years and older. Eligible participants were recruited consecutively and their responses were collected simultaneously. Participants received incentive points redeemable for cash, gift certificates, or mileage. The study protocol was approved by the Ethics Review Board of Okayama University Hospital (No. ken‐2405‐041). Participants were presented with an online consent statement at the beginning of the survey, and only those who completed the survey were included in the study. This study adhered to the Strengthening the Reporting of Observational Studies in Epidemiology (STROBE) guidelines [15].

### 2.2 Screening Process and Data Collection

Quality assurance measures to mitigate random variability and enhance data reliability have been described previously [5, 16]. Briefly, the survey employed multiple screening items to ensure the validity of responses. Participants were initially asked to select any non‐communicable diseases for which they had received treatment at least twice within the past 6 months, with the option of selecting multiple conditions. They were then asked to identify the most burdensome diseases based on their selections. Any inconsistencies between the diseases reported in these two items led to the exclusion of the participant's data. Furthermore, respondents were asked to list the medications prescribed for their most burdensome diseases. Any discrepancies between the most burdensome disease and appropriate medications for that disease resulted in exclusion. Two researchers independently verified the listed medications against an online database of label information and resolved any discrepancies by consensus to ensure consistency. An exception was made for respondents who selected cancer and reported “none” for prescribed medications, as the watch‐and‐wait approach is considered appropriate for certain cancer treatments. Additionally, a response time cut‐off of 300 s, determined through prior pilot testing, was applied to exclude overly quick responses that could indicate random or non‐serious answers.

### 2.3 Outcome

The primary outcomes were general trust in physicians and interpersonal trust in personal physicians. Trust was measured using the Japanese version of the 5‐item Wake Forest Physician Trust Scale: *Trust in Doctors Generally* and *Interpersonal Trust in Physician* [5, 17]. Each item was rated on a 5‐point Likert scale ranging from 1 (*totally disagree*) to 5 (*totally agree*). One negatively worded item was reverse coded, and the total score for each scale was normalized to a range of 0–100.

Before answering the *Interpersonal Trust in Physician* scale, respondents were instructed as follows: “Please think of the doctor who cares for your [the most troublesome disease chosen by the participants was automatically displayed here] when you answer these questions. He or she will be considered your doctor for this survey. For the next question, we are interested in your honest opinion about your doctor. Please choose the answer that best matches your opinion for each question.”

For the *Trust in Doctors Generally* scale, respondents received the following instructions: “The following questions may seem similar to the previous ones. However, they are not about your doctors, however, doctors in general. No need to be concerned if these issues have not been considered previously. No correct or incorrect responses were obtained. Please choose the answer that best matches your thoughts on doctors.”

### 2.4 Exposures

Exposure included three types of administrative errors: prescription errors, appointment scheduling omissions, and confidentiality breaches. Respondents were first provided with the following instructions: “Please select the response that best describes your opinion regarding the following statements about your doctor who cares for your [the most troublesome disease chosen by the participants was automatically displayed here]”. The following items were then presented: “Has your doctor ever failed to prescribe your medication, or mistakenly prescribed wrong types or dosages of your medication?”, “Has your doctor previously forgotten an outpatient or exam appointment?”, “Has your doctor ever previously discussed your illness or personal information with you in a place where someone you did not know could hear you?” For each item, respondents were asked to select either (1) (*have had*) or (2) (*have not*).

### 2.5 Covariates

Potential confounders were identified based on prior literature and clinical expertise. These included age [1, 18], sex [18], final education, household income [18], reported disease, duration of the patient‐physician relationship [1], and the patient's general level of interpersonal traits. The patients' general level of interpersonal trust was assessed using the 6‐item General Trust Scale [19], rated on a 5‐point Likert scale ranging from *strongly disagree* to *strongly agree*. The total score was computed by summing the responses to all items.

### 2.6 Statistical Analysis

Demographic and clinical variables are summarized as medians with interquartile ranges for continuous variables and frequencies with percentages for categorical variables. We used general linear models to estimate the associations between the three types of administrative errors and both interpersonal trust in a physician and general trust in physicians. Cluster‐robust variance estimation was used to account for the within‐prefecture clustering [20]. Furthermore, the effect modification by participants' reported diseases on the associations between administrative errors and the outcomes was evaluated using the regression models applied in the main analyses, additionally including an interaction term between each exposure and reported disease. To assess whether trust in personal physicians mediates the relationship between specific administrative errors and general trust in physicians, we conducted a mediation analysis. A series of mediation models were fitted to examine the hypothesized relationships between administrative errors, interpersonal trust (mediator), and general trust while adjusting for the previously described covariates. The mediation analysis was performed using the user‐written Stata command “sgmediation2” [21]. All statistical analyses were performed using Stata version 18 (StataCorp).

## 3 Results

### 3.1 Participant Characteristics

A total of 964 participants met the inclusion criteria; however, 247 were excluded due to inconsistent responses regarding their most troubling disease and medication, and 46 were excluded for responding too quickly to provide logical answers [5]. Of the remaining 671 eligible participants, 661 (mean age: 63 years; 26.5% female) were included in the primary analyses and 10 were excluded due to missing data on covariates. The baseline characteristics are shown in Table 1. The most common diseases were cancer (*n* = 255; 38.6%), diabetes mellitus (*n* = 191; 28.9%), and depression (*n* = 127; 19.2%). Among the participants, 92 (13.9%) reported experiencing at least one administrative error, 72 (10.9%) experienced a prescription error, 18 (2.7%) experienced a confidentiality breach, and 26 (3.9%) reported appointment scheduling omissions.

**TABLE 1:** Participants' characteristics.

Baseline characteristics	Total (*N* = 661)
Age, years	63.0 (56.0–70.0)
Male sex	486 (73.5%)
Education	
Junior high school	19 (2.9%)
High school	209 (31.6%)
Junior college	65 (9.8%)
University	325 (49.2%)
Graduate school	30 (4.5%)
Not answered	13 (2.0%)
Household income	
< 1,000,000 yen	40 (6.1%)
1,000,000 to < 3,000,000 yen	157 (23.8%)
3,000,000 to < 5,000,000 yen	203 (30.7%)
5,000,000 to < 10,000,000 yen	205 (31.0%)
≥ 10,000,000 yen	56 (8.5%)
Patient‐physician relationship duration	
< 1 years	60 (9.1%)
1 to < 3 years	212 (32.1%)
≥ 3 years	389 (58.9%)
General level of interpersonal trust	54.2 (45.8–70.8)
Reported diseases	
Arrhythmia	37 (5.6%)
Ischemic heart disease	119 (18.0%)
Heart failure	15 (2.3%)
Diabetes mellitus	191 (28.9%)
Rheumatoid arthritis/systemic lupus erythematosus	17 (2.6%)
Cancer	255 (38.6%)
Depression	127 (19.2%)
The most troublesome disease	
Arrhythmia	17 (2.6%)
Ischemic heart disease	89 (13.5%)
Heart failure	8 (1.2%)
Diabetes mellitus	175 (26.5%)
Rheumatoid arthritis/systemic lupus erythematosus	13 (2.0%)
Cancer	242 (36.6%)
Depression	117 (17.7%)

*Note:* Values are presented as medians (IQR) for continuous variables and *n* (%) for categorical variables.

### 3.2 Association Between Trust in Physicians and Three Types of Administrative Errors

**FIGURE 1:**
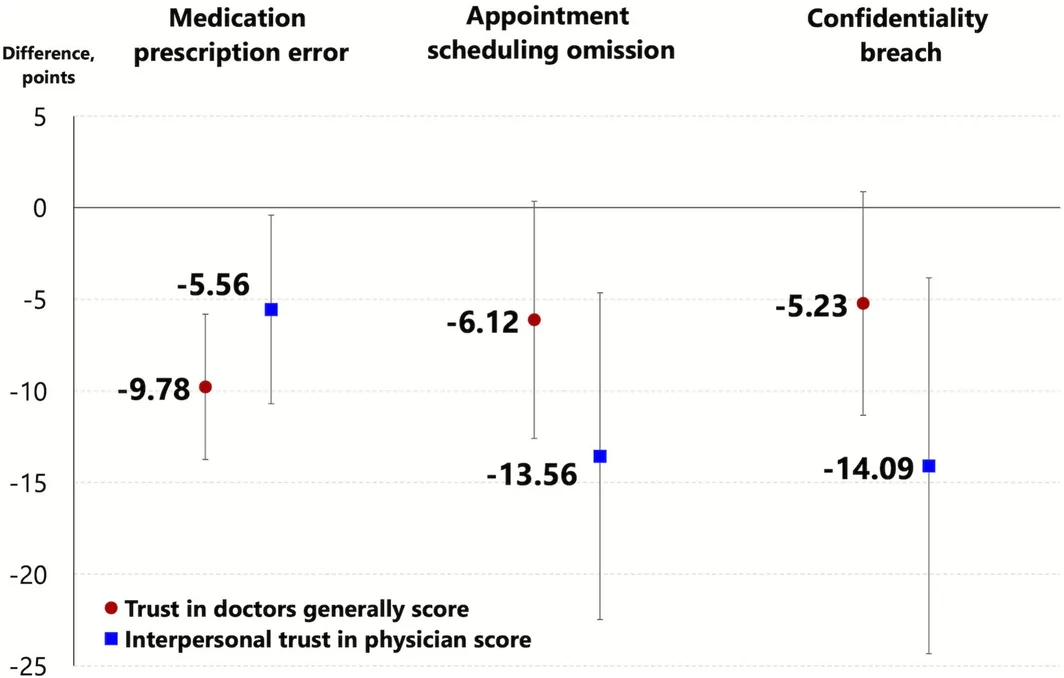
Association between trust in physicians and administrative errors. The general linear model described in the footnotes of Table 2 was fitted to each outcome. Red circles indicate the adjusted mean difference (coefficient) in trust in doctors generally scores associated with administrative errors. The blue square indicates the adjusted mean difference (coefficient) of interpersonal trust in physician scores associated with administrative errors. Error bars indicate 95% confidence intervals.

After adjusting for covariates, all three types of errors were associated with decreased interpersonal trust in their physicians. As shown in Table 2 and Figure 1, confidentiality breaches had the most significant negative impact on interpersonal trust scores (coefficient estimate: −14.09 points [95% confidence interval (CI)]: −24.35 to −3.83; *p* = 0.008), followed by appointment scheduling omissions (−13.56 points, 95% CI: −22.48 to −4.65; *p* = 0.004). Medication prescription errors also significantly reduced interpersonal trust (−5.56 points, 95% CI: −10.70 to −0.41; *p* = 0.035). In contrast, general trust in physicians was primarily associated with medication prescription errors, which led to a reduction of (9.78 points, 95% CI: −13.74 to −5.81; *p* < 0.001). No significant associations were found between general trust in physicians and appointment scheduling omissions (−6.12 points, 95% CI: −12.59 to 0.35; *p* = 0.063) or confidentiality breaches (−5.23 points, 95% CI: −11.33 to 0.88; *p* = 0.091).

**TABLE 2:** Association of administrative errors with trust in doctors generally and interpersonal trust in a physician.

	Interpersonal trust in a physician		Trust in doctors generally	
	Coefficient (95% CI)	*p*	Coefficient (95% CI)	*p*
Prescription error	−5.56 (−10.70 to −0.41)	0.035	−9.78 (−13.74 to −5.81)	< 0.001
Appointment scheduling omissions	−13.56 (−22.48 to −4.65)	0.004	−6.12 (−12.59 to 0.35)	0.063
Confidentiality breach	−14.09 (−24.35 to −3.83)	0.008	−5.23 (−11.33 to 0.88)	0.091

*Note:* The models were adjusted for baseline characteristics such as age, sex, final education, household income, duration of the patient‐physician relationship, reported comorbidities, and the patient's general interpersonal trust level. Standard errors were estimated using cluster‐robust variance, assuming that the prefectures were cluster units.

Abbreviation: CI, confidence interval.

### 3.3 Effect Modification by Reported Diseases on the Associations Between Trust in Physicians and Three Types of Administrative Errors

Most reported diseases did not significantly modify the associations between administrative errors and trust in physicians. However, certain patterns were observed: prescription errors among participants with ischemic heart disease, appointment scheduling omissions among those with arrhythmia or ischemic heart disease, and confidentiality breaches among those with rheumatoid arthritis/systemic lupus erythematosus seemed to have a greater negative impact on interpersonal trust in a physician compared to their counterparts (Table S1). Furthermore, the reduction of trust in doctors generally associated with prescription errors or appointment scheduling omissions was more pronounced among participants with depression and arrhythmia, respectively.

### 3.4 The Mediating Role of Trust in Their Own Physician in the Association Between Medication Prescription Error and Trust in General Physicians

**FIGURE 2:**
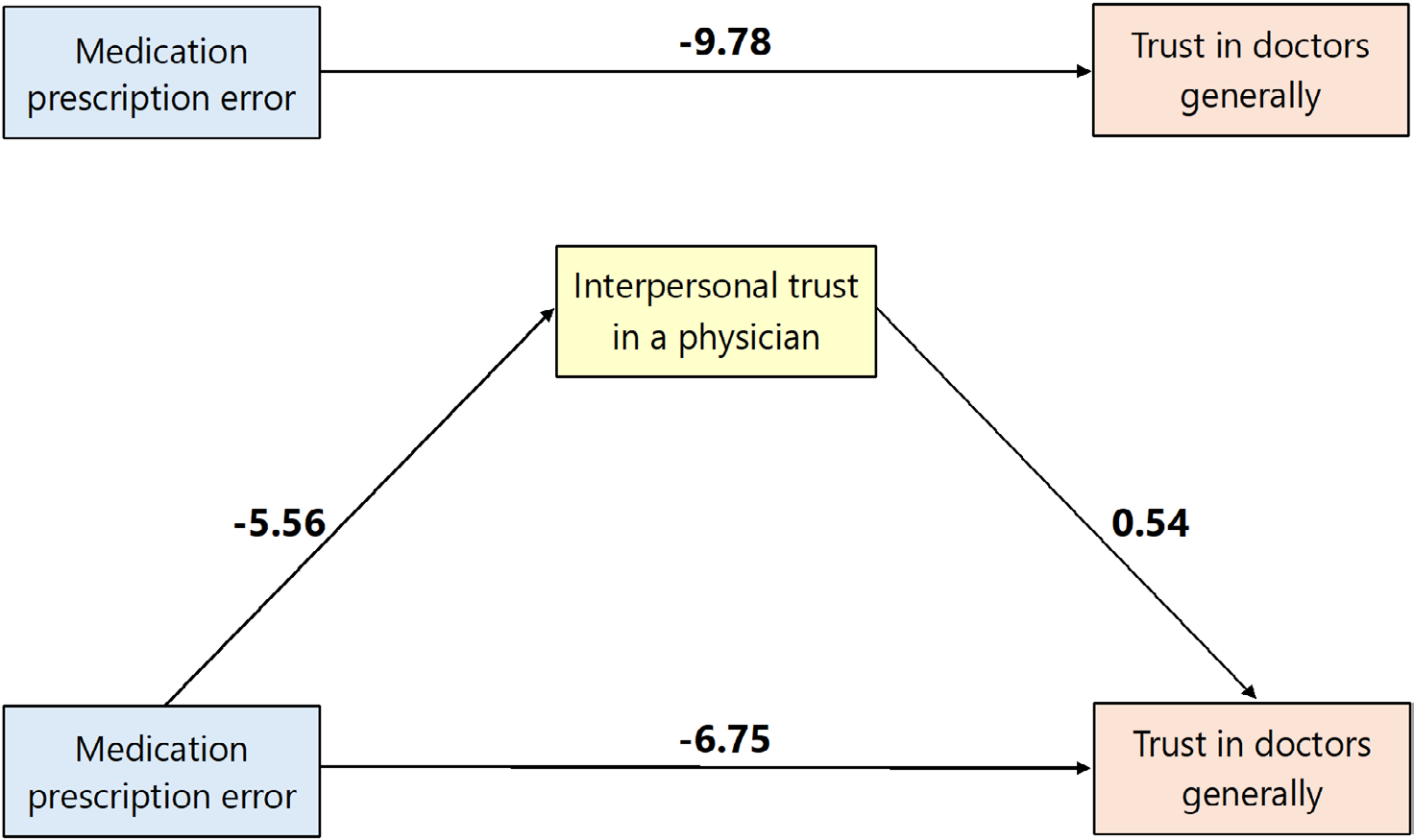
Mediating role of interpersonal trust in the association between medication prescription errors and general trust in doctors. This figure shows the direct and indirect paths between medication prescription errors and general trust in doctors, mediated by interpersonal trust in a physician. Direct effect: −6.75, indirect effect: −3.02 (Sobel test, *p* = 0.031). The total effect of medication errors on general trust is −9.78, with 30.9% of this effect mediated through interpersonal trust in the attending physician.

Medication prescription errors were the only type of administrative error significantly associated with decreased general trust in physicians. Both direct effects (69% of the total effect) and indirect effects (31% of the total effect) of medication prescription errors on general trust in physicians were observed. Mediation analysis revealed that a decline in interpersonal trust in their physicians partially mediated the relationship between medication prescription errors and the overall decline in general trust in physicians (Sobel test, *p* = 0.031; see Figure 2).

## 4 Discussion

This study underscores the significant impact of administrative errors on patient trust in physicians among individuals with non‐communicable diseases such as cancer, diabetes, and depression. Prescription errors were associated with reduced trust in both personal physicians and physicians in general, with the loss of trust in personal physicians partially mediating the loss of trust in general physicians.

Confidentiality breaches and omitted appointment scheduling by participants' physicians resulted in the greatest reduction in interpersonal trust scores, highlighting that ensuring reliable appointment scheduling is as crucial as maintaining patient confidentiality.

The reported incidence of prescription errors in primary care outpatient services affiliated with an academic medical center in the United States is 7.6% [22]. In comparison, the prevalence of prescription errors in this study was 10.9%, focusing specifically on missed prescriptions or errors in type or dosage. One reason our percentage was slightly higher than that of the previous study is that our study relied on patient‐reported reflections over a longer recall period, whereas the prior study used short‐term prospective verification of prescription records [22]. Few studies have comprehensively examined the loss of trust in physicians owing to prescription errors including their effects on general trust. Prior research quantified trust in physicians using vignettes involving adverse medication events; however, that study assessed trust based on disclosure practices rather than the presence of the error itself [23]. In this study, the reduction in trust in one's physician due to prescription errors (5.6 points) was comparable to the impact of misdiagnosis by a different physician (4.3 points) [16]. This finding suggests that prescription errors, which may be harmless [9], are often an unexpected experience, causing negative and persistent psychological consequences. Additionally, our findings on the loss of trust in general physicians stemming from prescription errors by their personal physicians support the argument that trust in general physicians is influenced to some extent by individual experiences with their personal physicians [4, 5]. In other words, prescription errors can erode trust not only in specific physicians but also in the broader healthcare system. Furthermore, the results highlighted that the loss of trust in general physicians due to prescription errors was partially mediated by diminished trust in one's own physicians. On the other hand, the direct loss of trust in general physicians attributable to prescription errors may be shaped by the societal perceptions of physicians conveyed through the media and other social channels [4, 5, 24]. Particularly, exaggerated portrayals of medical errors in television dramas may contribute to negative public perceptions of healthcare systems [24]. For example, an analysis of medical errors depicted in six episodes from each of eight popular U.S. medical television series (1994–2018) revealed that medical errors occurred at a rate of 6.4 times per hour, with medication errors being the third most common [24].

The findings indicate that, among patients with non‐communicable diseases requiring ongoing management, the loss of interpersonal trust resulting from physicians discussing patient illnesses or personal information in a way that can be overheard by others, is greater than that caused by prescription errors. The issue of healthcare professionals discussing patient information in institutional spaces with limited privacy has been studied in many countries. For instance, a study conducted in five U.S. hospitals found that approximately 14% of elevator rides involved inappropriate comments from healthcare professionals. The majority of confidentiality violations, including graphic discussions of patient identifiers and treatment plans, were attributed to physicians [25]. However, breaches of confidentiality stemmed from patients' perceptions of insufficient auditory privacy during interactions with their physicians. This issue can be attributed not only to physicians' attitudes toward confidentiality but also to the design of consultation rooms, a problem that may be specific to Japan. Consultation rooms in Japan are often not fully walled off on all four sides. In particular, the side opposite the patient entrance is frequently left open to facilitate the movement of medical staff between consultation rooms. This design flaw allows conversations to be overheard by patients in adjacent rooms. Similar issues have been reported in a U.S. emergency room study where patients often overheard discussions about themselves and others due to insufficient space and inadequate walls on all sides [26]. Another potential source of confidentiality breaches, one not unique to Japan, may involve ward rounds during past hospitalizations, where sensitive patient information was overheard by others in neighboring beds [11]. Unlike prescription errors, breaches of confidentiality did not significantly affect general trust in physicians in this study. This finding suggests that such breaches are viewed by patients as isolated incidents attributable to specific physicians rather than reflective of the healthcare system as a whole [12].

The loss of trust in physicians resulting from appointment scheduling omissions due to physicians' forgetting may be a unique issue in Japan. While follow‐up appointments are commonly scheduled through electronic healthcare records in many countries [27], this task is typically performed by the physicians themselves at the end of consultations in Japan. In contrast, in the U.S. and other countries, appointment scheduling is often handled by a hospital administrator [10]. Although clerical errors leading to omitted appointments are occasionally reported globally, the administrative burden on Japanese physicians may contribute to oversights in appointment scheduling. Such errors are often discovered only when patients inform their physicians. The magnitude of the loss of trust in personal physicians associated with omitted appointments, comparable to that of confidentiality breaches, suggests that patients attribute these administrative errors to their personal physician rather than to systemic issues.

This study has several strengths. First, we quantified the impact of specific categories of administrative errors on trust in both one's personal physician and general physicians, offering valuable insights into the dynamics of patient trust within healthcare settings. Second, by adjusting for patients' general tendency to trust others as a covariate, the analysis isolated the impact of these errors on trust in physicians independently of general trustworthiness.

However, this study has certain limitations, which must be acknowledged. First, recall bias may have been present, as the classification of administrative errors relied on participants' self‐reports, potentially leading to misclassification. For instance, confidentiality breaches may have been underestimated due to the reliance on participants' recollections of past interactions. Similarly, prescription errors and appointment scheduling omissions with minimal impact on clinical practice may also have been underreported. Second, the study did not examine the specifics of medical errors such as the type of medication error (e.g., dosage exceeding label instructions or misprescription of a medication with a different indication) or the consequences of these errors (e.g., the presence or absence, or severity of adverse events). Third, selection bias may have arisen from the use of an incentivized online panel survey. Participants with access to such panels tended to have higher educational levels and IT literacy, which may limit the representativeness of the target population. However, the direction of this potential bias in the estimates remains uncertain. Fourth, random errors may have occurred due to the relatively small sample size. Although most analyses had adequate statistical power to detect associations, the analyses examining the associations between appointment scheduling omissions and trust in doctors generally, and between confidentiality breach and trust in doctors generally were underpowered (post hoc power: 0.69 and 0.49, respectively), as estimated using a two‐sample *t*‐test (Table S2). The upper bounds of the 95% CIs in both analyses were close to the null, suggesting that larger sample sizes might have revealed associations between these administrative errors and trust in doctors generally. Fifth, heterogeneity induced by reported diseases may be a concern, given the observed effect modification by several diseases. However, most analyses stratified by reported diseases were not statistically significant. Although the sample size within each stratum was limited, this heterogeneity is unlikely to have affected the overall study conclusion.

In conclusion, this study provides unique insights into how administrative errors such as prescription errors, appointment scheduling omissions, and confidentiality breaches, directly and negatively impact the quality of physician‐patient interactions. Addressing these issues is crucial for fostering strong physician‐patient relationships and improving outcomes, particularly in the long‐term management of Japanese adults with non‐communicable diseases.

## Author Contributions

Tetsuro Aita, Yoshia Miyawaki, and Noriaki Kurita had full access to all data in the study and took responsibility for the integrity of the data and the accuracy of the data analysis. Concept and design: Tetsuro Aita, Noriaki Kurita. Acquisition, analysis, or interpretation of data: All authors. Drafting of the manuscript: Tetsuro Aita, Yoshia Miyawaki, Noriaki Kurita. Critical revision of the manuscript for important intellectual content: All authors. Statistical analysis: Tetsuro Aita, Yoshia Miyawaki, Noriaki Kurita. Obtained funding: Noriaki Kurita. Administrative, technical, or material support: Takafumi Wakita, Nobuyuki Yajima. Supervision: Nobuyuki Yajima, Takafumi Wakita, Ashwin B. Gupta.

## Funding

This study was supported by JSPS KAKENHI, Grant Numbers 19KT0021 (N.K.) and 22K19690 (N.K.).

## Ethics Statement

The study protocol was approved by the Ethics Review Board of Okayama University Hospital (No. ken‐2405‐041).

## Conflicts of Interest

The authors declare no conflicts of interest.

## Supporting information


**Table S1:** The effect modification by participants' reported diseases on the association between administrative errors and trust in physicians, both general and interpersonal.
**Table S2:** Post hoc statistical power calculated based on the obtained outcome means and standard deviations, and the number of samples.

## Data Availability

The data that support the findings of this study are available from the corresponding author upon reasonable request.
