# Supplementary material for: The Effect of Three Key Administrative Errors on Patient Trust in Physicians: Prescription Errors, Confidentiality Breaches, and Appointment Scheduling Omissions
Source: J Gen Fam Med. 2025 Nov 30;27(1):e70086. doi: 10.1002/jgf2.70086 (PMC12897576; doi:10.1002/jgf2.70086)
Supplement: Supplementary file 1 — Table S1: The effect modification by participants' reported diseases on the association between administrative errors and trust in physicians, both general and interpersonal. Table S2: Post hoc statistical power calculated based on the obtained outcome means and standard deviations, and the number of samples. [file JGF2-27-e70086-s001.docx]

Supplementary Table S1. The effect modification by participants’ reported diseases on the association between administrative errors and trust in physicians, both general and interpersonal.

|  |  | Interpersonal trust in a physician | | Trust in doctors generally | |
| --- | --- | --- | --- | --- | --- |
| Error | Reported diseases | Coefficient (95% CI) | P for interaction | Coefficient (95% CI) | P for interaction |
| Prescription errors |  |  |  |  |  |
|  | Arrhythmia |  |  |  |  |
|  | No | –6.00 (–11.15 to –0.85) | 0.191 | –9.47 (–13.87 to –5.07) | 0.279 |
|  | Yes | 2.07 (–10.55 to 14.69) |  | –15.07 (–22.29 to –7.85) |  |
|  | Ischemic heart disease |  |  |  |  |
|  | No | **–4.43 (–9.98 to 1.12)** | **0.022** | –9.99 (–14.21 to –5.76) | 0.535 |
|  | Yes | **–19.70 (–32.84 to –6.55)** |  | –6.86 (–15.35 to 1.63) |  |
|  | Heart failure |  |  |  |  |
|  | No | NA |  | NA |  |
|  | Yes | NA |  | NA |  |
|  | Diabetes mellitus |  |  |  |  |
|  | No | –6.77 (–11.80 to –1.73) | 0.222 | **–12.39 (–16.60 to –8.19)** | **0.021** |
|  | Yes | –2.42 (–10.32 to 5.48) |  | **–2.95 (–9.91 to 4.02)** |  |
|  | RA/SLE |  |  |  |  |
|  | No | –4.97 (–10.17 to 0.23) | 0.056 | –9.62 (–13.69 to –5.54) | 0.65 |
|  | Yes | –19.00 (–32.37 to –5.63) |  | –12.95 (–26.95 to 1.05) |  |
|  | Cancer |  |  |  |  |
|  | No | –6.38 (–11.92 to –0.84) | 0.704 | –7.41 (–11.68 to –3.13) | 0.15 |
|  | Yes | –4.50 (–13.35 to 4.34) |  | –12.73 (–18.92 to –6.54) |  |
|  | Depression |  |  |  |  |
|  | No | –4.82 (–10.19 to 0.56) | 0.416 | **–8.23 (–12.83 to –3.63)** | **0.035** |
|  | Yes | –8.16 (–16.99 to 0.68) |  | **–15.12 (–21.02 to –9.22)** |  |
| Appointment scheduling omissions |  |  |  |  |  |
|  | Arrhythmia |  |  |  |  |
|  | No | **–12.80 (–21.80 to –3.81)** | **0.003** | **–5.53 (–12.04 to 0.98)** | **0.021** |
|  | Yes | **–30.17 (–40.81 to –19.52)** |  | **–19.09 (–30.71 to –7.46)** |  |
|  | Ischemic heart disease |  |  |  |  |
|  | No | **–8.82 (–18.08 to 0.45)** | **< 0.001** | –5.62 (–12.29 to 1.06) | 0.606 |
|  | Yes | **–34.34 (–43.40 to –25.29)** |  | –8.21 (–18.87 to 2.44) |  |
|  | Heart failure |  |  |  |  |
|  | No | NA |  | NA |  |
|  | Yes | NA |  | NA |  |
|  | Diabetes mellitus |  |  |  |  |
|  | No | –12.42 (–24.04 to –0.80) | 0.563 | –6.32 (–13.24 to 0.60) | 0.792 |
|  | Yes | –17.56 (–27.96 to –7.16) |  | –5.33 (–13.76 to 3.10) |  |
|  | RA/SLE |  |  |  |  |
|  | No | –13.36 (–22.51 to –4.22) | 0.885 | –6.88 (–13.36 to –0.41) | 0.575 |
|  | Yes | –15.35 (–42.17 to 11.47) |  | 1.03 (–26.12 to 28.18) |  |
|  | Cancer |  |  |  |  |
|  | No | –22.07 (–35.76 to –8.37) | 0.167 | –7.90 (–18.10 to 2.29) | 0.629 |
|  | Yes | –8.38 (–20.12 to 3.37) |  | –5.00 (–12.57 to 2.56) |  |
|  | Depression |  |  |  |  |
|  | No | –14.44 (–23.78 to –5.10) | 0.507 | –4.85 (–11.43 to 1.74) | 0.512 |
|  | Yes | –8.60 (–26.03 to 8.83) |  | –13.11 (–37.32 to 11.11) |  |
| Confidentiality breaches |  |  |  |  |  |
|  | Arrhythmia |  |  |  |  |
|  | No | –14.19 (–26.08 to –2.29) | 0.922 | –4.41 (–11.62 to 2.81) | 0.313 |
|  | Yes | –13.58 (–19.94 to –7.22) |  | 9.59 (–16.48 to –2.69) |  |
|  | Ischemic heart disease |  |  |  |  |
|  | No | –14.19 (–24.16 to –4.23) | 0.893 | –4.60 (–11.65 to 2.47) | 0.718 |
|  | Yes | –12.97 (–32.83 to 6.90) |  | –6.14 (–11.62 to –0.65) |  |
|  | Heart failure |  |  |  |  |
|  | No | NA |  | NA |  |
|  | Yes | NA |  | NA |  |
|  | Diabetes mellitus |  |  |  |  |
|  | No | –13.76 (–28.92 to 1.40) | 0.943 | **–9.87 (–18.82 to –0.93)** | **0.05** |
|  | Yes | –14.37 (–23.15 to –5.58) |  | **3.02 (–4.44 to 10.48)** |  |
|  | RA/SLE |  |  |  |  |
|  | No | **–13.11 (–22.78 to –3.44)** | **0.011** | –4.65 (–11.04 to 1.74) | 0.573 |
|  | Yes | **–30.34 (–41.41 to –19.27)** |  | –8.41 (–17.96 to 1.14) |  |
|  | Cancer |  |  |  |  |
|  | No | –18.17 (–27.97 to –8.38) | 0.428 | –3.82 (–9.21 to 1.57) | 0.769 |
|  | Yes | –7.18 (–31.43 to 17.07) |  | –6.51 (–22.52 to 9.49) |  |
|  | Depression |  |  |  |  |
|  | No | –12.90 (–25.09 to –0.71) | 0.397 | –3.71 (–10.55 to 3.13) | 0.341 |
|  | Yes | –19.32 (–28.34 to –10.30) |  | –10.33 (–21.33 to 0.67) |  |

Note: The models were adjusted for baseline characteristics such as age, sex, final education, household income, duration of the patient-physician relationship, reported comorbidities, and patient’s general interpersonal trust level. Standard errors were estimated using cluster-robust variance, assuming that the prefectures were cluster units. Additionally, each model incorporated an interaction term between the exposure and the reported disease. The Wald tests were examined to obtain p-values for interaction. Abbreviations: CI, confidence interval; NA, not available; RA, Rheumatoid arthritis; SLE, Systemic lupus erythematosus

Supplementary Table S2. Post-hoc statistical power calculated based on the obtained outcome means and standard deviations, and the number of samples

| Exposure | Outcome | Study power |
| --- | --- | --- |
| Prescription error | Interpersonal trust in a physician | 0.9084 |
|  | Trust in doctors generally | 0.9912 |
| Appointment scheduling omission | Interpersonal trust in a physician | 0.8347 |
|  | Trust in doctors generally | 0.6887 |
| Confidentiality breach | Interpersonal trust in a physician | 0.8282 |
|  | Trust in doctors generally | 0.4893 |

Note: Post-hoc power was estimated using a two-sample t-test (α = 0.05), derived from the observed outcome means and standard deviations, and the number of participants in each group.
